# Minimally Invasive Isolated and Hybrid Surgical Revascularization for Multivessel Coronary Disease: A Single-Center Long-Term Follow-Up

**DOI:** 10.3390/jpm14050528

**Published:** 2024-05-15

**Authors:** Tiziano Torre, Alberto Pozzoli, Marco Valgimigli, Laura Anna Leo, Francesca Toto, Mirko Muretti, Sara Birova, Enrico Ferrari, Giovanni Pedrazzini, Stefanos Demertzis

**Affiliations:** 1 Heart Surgery Unit, Cardiocentro Ticino Institute, EOC, 6900 Lugano, Switzerland; tiziano.torre@eoc.ch (T.T.); francesca.toto@eoc.ch (F.T.); mirko.muretti@eoc.ch (M.M.); sara.birova@eoc.ch (S.B.); enrico.ferrari@eoc.ch (E.F.); stefanos.demertzis@eoc.ch (S.D.); 2 Cardiology Unit, Cardiocentro Ticino Institute, EOC, 6900 Lugano, Switzerlandlauraanna.leo@eoc.ch (L.A.L.); giovanni.pedrazzini@eoc.ch (G.P.); 3 Faculty of Biomedical Sciences, Università della Svizzera Italiana (USI), 6900 Lugano, Switzerland; 4 Faculty of Medicine, University of Zurich (UZH), 8032 Zurich, Switzerland; 5 Faculty of Medicine, University of Bern, 3010 Bern, Switzerland

**Keywords:** multivessel coronary disease, minimally invasive coronary bypass surgery, MIDCAB, beating-heart coronary surgery, hybrid coronary revascularization, percutaneous coronary intervention, heart team

## Abstract

Introduction: Some evidence suggests that surgical minimally invasive (MIDCAB) and hybrid coronary revascularization (HCR) are safe and potentially effective at short-term follow-up. Data on long-term outcomes are more limited and inconclusive. Methods: Between February 2013 and December 2023, a total of 1997 patients underwent surgical coronary artery revascularization at our institution, of whom, 92 (4.7%) received left anterior mini-thoracotomy access (MIDCAB), either isolated (N = 78) or in combination with percutaneous coronary intervention (N = 14, HCR group). Results: After a median follow-up of 75 months (range 3.1: 149 months), cardiac mortality was 0% while overall mortality was 3%, with one in-hospital mortality and two additional late deaths. Conversion to sternotomy happened in two patients (2.1%), and surgical re-explorations occurred in five patients (4.6%), of whom three for bleeding and two for graft failure. All patients received left internal mammary (LIMA) to left anterior descending artery (LAD) grafting (100%). In the HCR group, 10 patients (72%) showed percutaneous revascularization (PCI) after MIDCAB, showing PCI on a mean of 1.6 ± 0.6 vessels and implanting 2.1 ± 0.9 drug-eluting stents. Conclusions: MIDCAB, in isolation or in association with hybrid coronary revascularization, is associated with encouraging short- and long-term results in selected patients discussed within a dedicated heart-team.

## 1. Introduction

Coronary artery bypass grafting (CABG) is the most common adult surgery procedure performed globally as well as the foundation of cardiac surgery, which has evolved considerably since the time of its introduction approximately 50 years ago [1].

**Figure 1:**
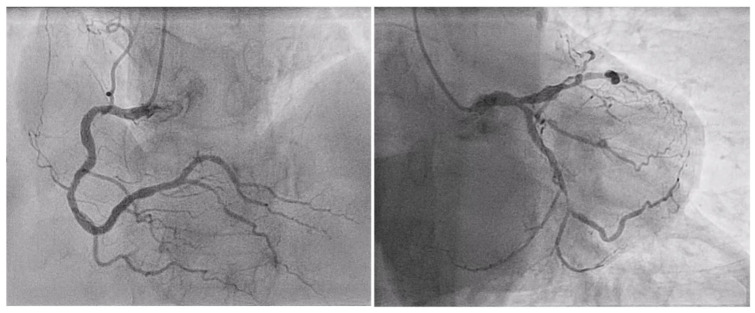
Preoperative coronary angiogram of an 80-year-old patient suffering from multivessel coronary disease on the distal right coronary artery (**left**) and diffusely on the left coronary system (**right**).

**Figure 2:**
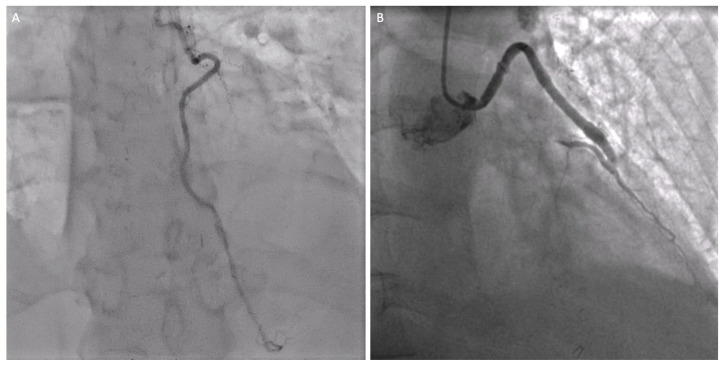
Postoperative coronary angiogram after bypass surgery performed in a minimally invasive fashion on the left anterior descending (LAD) with the left internal mammary artery (LIMA) (**A**) and the angiographic result of the venous graft on the diagonal branch (**B**).

**Figure 3:**
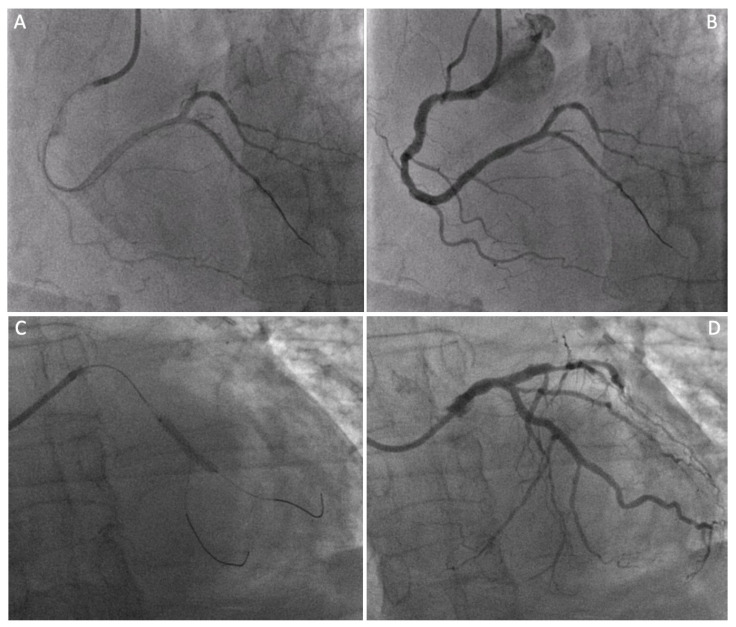
Four panel figure depicting the PCI treatment with stenting of the non-LAD vessels. Panel (**A**,**B**): angiographic result after percutaneous revascularization (PCI) with a drug-eluting stent on the distal portion of the right coronary artery. Panel (**C**,**D**): angiographic result after percutaneous revascularization (PCI) with a drug-eluting stent at the obtuse marginals’ bifurcation of the circumflex coronary artery.

Minimally invasive direct coronary artery bypass (MIDCAB) grafting has been suggested as an effective and less invasive alternative to traditional CABG for the revascularization of the left anterior descending artery [2,3]. Over the last twenty years, the aim to achieve more extensive revascularization, maintaining a minimally invasive approach, has led many experienced surgeons to treat multivessel disease, too [4]. The results of the Syntax (Synergy Between Percutaneous Coronary Intervention with Taxus and Cardiac Surgery) Trial in 2014 clearly showed that surgery is the gold standard for three-vessel coronary disease, especially for those individuals with complex anatomies. Surgical revascularization was shown to be superior to percutaneous coronary intervention (PCI) with a first-generation drug-eluting stent (DES) with respect to the composite endpoint of death, myocardial infarction, stroke, and repeated revascularization [5]. On the other hand, a high rate of saphenous vein graft failures, sternal complications, and bleeding events have been observed in CABG patients. The routine use of second-generation DES was subsequently shown to be associated with a lower rate of restenosis and thrombosis than saphenous graft failure [6,7,8]. These results led, in 2018, to a statement of the European guidelines for CABG to be the standard treatment for multivessel disease (Figure 1), when a left internal mammary artery (LIMA) to left anterior descending (LAD) grafting is performed. Since the new generation DES provides satisfactory short- and long-term clinical outcomes, the armamentarium would be considered high quality on both arms [7]. In fact, saphenous vein grafts (SVG) exhibit lower patency and a higher mortality rate compared with those of LIMA. The SVGs have been shown to occlude (up to 50%) as early as 10 years after implantation due to many factors [8]. The failure of these grafts reached a rate of 30% to 40% after 10 years, due to patients developing SVG intimal hyperplasia. In addition to intimal thickening, SVGs can undergo atherosclerosis, with angiographic studies demonstrating an attrition rate of the SVG of 2% from the first to the seventh post-operative year with only 38% to 45% of SVGs remaining patent after 10 years [8]. Hence, the introduction in clinical practice of a hybrid coronary revascularization (HCR) to deal with multivessel coronary artery disease has been based on the wish to combine the best of the two therapies (Figure 2 and Figure 3). In 2011, the Guidelines of the American College of Cardiology (ACC) recommended a Class II a for hybrid revascularization, indicated only in selected patients not otherwise approachable with traditional surgical or percutaneous revascularization [9]. However, there are only a few randomized trials in the literature supporting the evidence, one of which was prematurely discontinued for suboptimal enrollment, which led to inconclusive recommendations from the most recent Guidelines [10]. Due to the lack of robust evidence supporting the HCR strategy, our daily clinical practice could only be based on the studies comparing HCR to traditional CABG or PCI. The aim of this original article is to report the outcomes of our institutional program on isolated and hybrid minimally invasive coronary surgery. Furthermore, the strategies adopted within the Heart Team according to the Guidelines will be discussed, with respect to the indication, timing, and staging of the two procedures.

## 2. Materials and Methods

### 2.1. Study Design

Between February 2013 and December 2023, a total of 1997 patients with coronary artery disease were referred for CABG at our Institution. Within this study period, MIDCAB and HCR patients were extracted. Baseline patient characteristics and surgical details were prospectively collected in the institutional electronic medical database and retrospectively analyzed for this study. The study was conducted according to the privacy policy of the Cardiocentro Ticino Institute and the internal regulations for the appropriate use of anonymized data in patient-oriented research, which are based on international regulations, including the Declaration of Helsinki (MEC-2020-0454). All patients signed informed consent forms for surgery and the Ethics Committee of Canton of Ticino approved the study design (CE 3103/BASEC 2016-0519, approval date: 22 March 2017). The follow-up was conducted either by analyzing the hospital medical records or via telephone calls to the patients or the general practitioners. In case of no response, we questioned the national mortuary office. The follow-up ended in December 2023.

### 2.2. Inclusion and Exclusion Criteria for MIDCAB /HCR and Pharmacologic Strategy

All patients were discussed within the Heart Team, either for isolated MIDCAB surgery or MIDCAB combined with percutaneous treatment of non-LAD vessels for a hybrid revascularization strategy, according to the international Guidelines [10].

**Figure 4:**
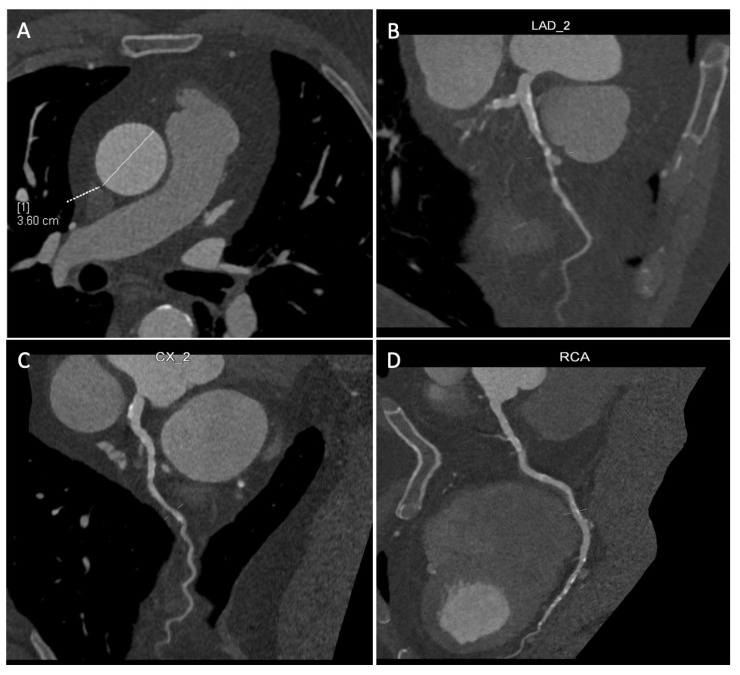
Coronary multislice computed tomography (MSCT) performed before the operation, with the analysis of the ascending aorta (**A**) and of the three epicardial coronaries (**B**–**D**), including the left main. [1] It refers to the calculated diameter of the ascending aorta (white dashed and solid lines).

To perform MIDCAB surgery at our institution, a combination of multiple inclusion criteria was mandatory, divided into three different domains, namely, anatomical, physiological, and surgical:

- Favorable chest anatomy to properly expose the heart and absence of calcifications or obstructing plaques of the femoral arteries, in case a peripheral cannulation for the cardiopulmonary bypass, would be needed.
- Hemodynamic stability and an adequate pulmonary function to tolerate single lung ventilation.
- Absence of calcification of the ascending aorta, allowing the execution of the proximal anastomosis (Figure 4A).

Absolute contraindications to MIDCAB at our institution are severe chest wall deformities (e.g., pectus excavatum), severe lung pathologies, and, obviously, emergent surgery with hemodynamic instability.

The MIDCAB was preferentially performed as a first-stage procedure within an HCR strategy and followed by PCI on the other vessels whenever feasible. Instead, culprit lesions were treated percutaneously during the same hospitalization or, if clinically feasible, in a new hospitalization at least one month after discharge. One hundred milligrams of acetylsalicylic acid was administered before the operation and continued indefinitely. The antiplatelet regimen included a loading dose of clopidogrel 300 mg or ticagrelor 180 mg at the time of PCI, with a recommended treatment duration of 12 months.

In patients with acute coronary syndrome, the culprit lesion on the non-LAD vessel is treated immediately and the surgical revascularization on the LAD is staged thereafter. Other non-LAD vessels were scheduled based on the severity of the lesions. In these patients, the P2Y12 receptor inhibitor was discontinued 3 to 5 days before the admission and/or surgery, and intravenous Cangrelor was used to embricate the P2Y12 receptor inhibitor. Every case is evaluated by the Multiplate^®^ Analyzer (Roche, Rotkreuz, Switzerland) to test the platelet function and confirm the operability. The DAPT therapy was to be reintroduced on the second postoperative day and continued according to individual bleeding and ischemic risks.

### 2.3. Preoperative Planning

**Figure 5:**
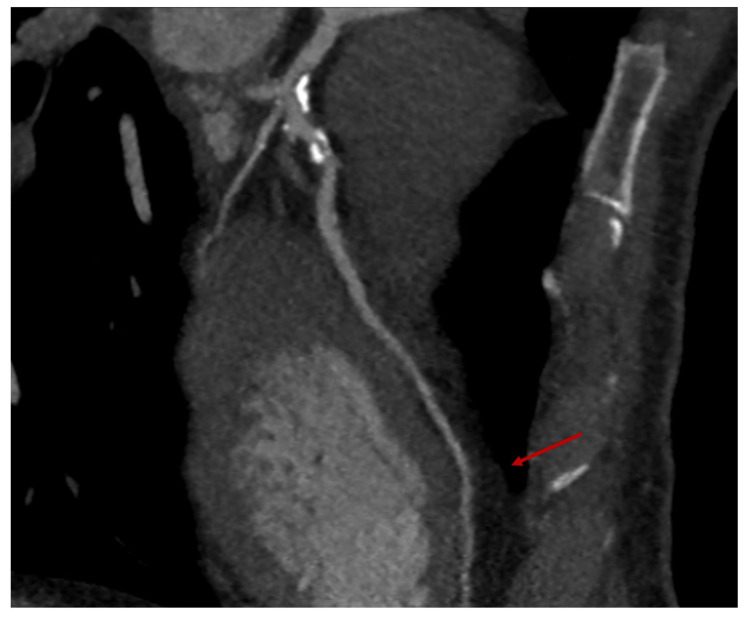
The coronary multislice computed tomography (MSCT), depicting—red arrow—the intramyocardial course of the left anterior descending (LAD), which represents a fundamental exclusion criteria prior to minimally invasive coronary surgery (MIDCAB).

All patients undergoing MIDCAB intervention, beyond coronary angiogram and a baseline transthoracic echocardiography, require further imaging with a cardiac multislice computed tomography (MSCT) with 3D reconstruction of the target vessels (left anterior descending artery particularly, then the diagonal or lateral/posterolateral branches) to exclude an intra-myocardial course, identify the target zone for the coronary anastomosis, and analyze the ascending aorta (Figure 4A–D and Figure 5).

### 2.4. Surgical Technique

**Figure 6:**
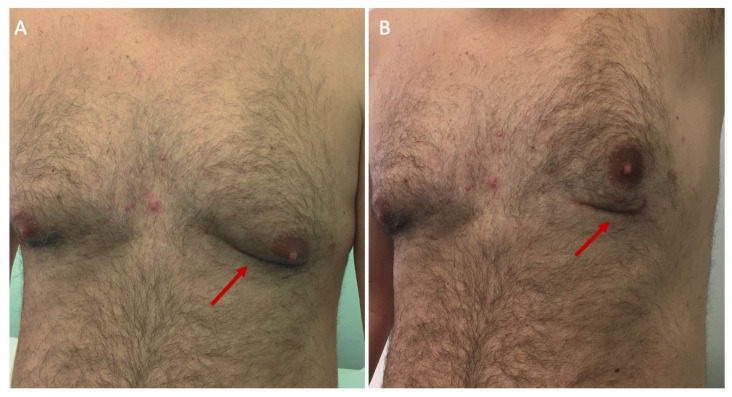
Postoperative cosmetic result after minimally invasive bypass surgery, implanting the left internal mammary artery on the left anterior descending. The reduced scar is visible (red arrows) in the submammary crease with the left arm at rest (**A**) and with the left arm raised (**B**).

All patients were positioned in a semi-supine position and the left chest was elevated at 30°. General mixed anesthesia was induced. An invasive arterial blood pressure monitoring (preferably right radial artery) was obtained. The intubation was performed with the use of a double-lumen endotracheal tube. A central venous port was introduced through the right jugular vein. A Foley catheter was passed into the bladder. Transesophageal echocardiography (TEE) was adopted in every patient. The groins were prepared in case of hemodynamic instability needing cardiopulmonary bypass. A left anterolateral mini-thoracotomy of 5 to 7 cm was performed in the sub-mammary crease according to the particular case. Since 2020, depending on the incision and procedure, a combination of intercostal, pectoralis, and serratus anterior nerve blockades have been adopted. The skin in the crease was marked the day before the operation, with the patient carefully seated on the bed and the pectoral muscles relaxed. In the operating room, the left internal mammary artery (LIMA) was harvested in a skeletonized fashion and under direct vision through the fourth intercostal space. At the beginning of our experience in 2013, we adopted the MIDAccess IMA retractor system (Delacroix-Chevalier, Paris, France) and starting in 2019, the MICS CABG Fehling retractor (Fehling Instruments, Karlstein am Main, Germany), which allowed either the left and the right internal thoracic artery harvesting. After opening and suspending the pericardium in a circular fashion, the left anterior descending (LAD) was identified, along with its target point of anastomosis, to check the effective length of the grafts. Heparin was administered in a standard dose of 10.000 UI to achieve an activated clotting time > 250 s. A disposable suction Medtronic Octopus stabilizer (Medtronic Inc., Minneapolis, MI, USA) was routinely adopted. In case of difficult exposure of the targeted vessels, including the lateral and inferior walls, a transthoracic stabilizer was favored (Nuvo, Medtronic Inc., Minneapolis, MI, USA). For the distal anastomosis, once the epicardium was stabilized, a blower was adopted to visualize the anastomotic area, and an intracoronary shunt was always inserted into the artery. The anastomotic suture was usually executed with a polypropylene 8-0 monofilament, with standard coronary instruments. In case proximal anastomosis or aortic cross-clamping were required, the ascending aorta was encircled with a Dacron tape, in order to freely mobilize it and enhance the exposure. In the case of hemodynamic instability or in the case of suboptimal target vessel exposure, the cardiopulmonary bypass could be instituted via peripheral femoral vessels. Every coronary conduit was assessed with a flow probe based on TransitTime Flow Measurement (TTFM) by Medistim ASA (Oslo, Norway) at the end. At chest closure, one Blake 24 Fr soft drain was routinely placed in the left pleural space via the 6th intercostal incision. Usually, the left lung has been gently re-inflated to avoid damage to the graft(s). The two ribs were normally tied together with a 2-0 Vicryl-coated suture. The skin closure was performed with 4-0 STRATAFIX™ Spiral Knotless (Figure 6). Postoperative analgesia was enhanced by slow a continuous anesthetic delivery of bupivacaine of 5% through small catheters positioned inside the wound.

### 2.5. Statistical Analysis

The statistical analysis was performed using Stata 17 (StataCorp, College Station, TX, USA). Continuous variables are presented as mean ± standard deviation and categorical variables are presented as numbers and percentages.

## 3. Results

Between February 2013 and December 2023, a total of 1997 patients with coronary artery disease were referred for CABG at our institution. Ninety-two of them (4.6%) were operated on through a left anterior mini-thoracotomy access. In the period from January 2017 to November 2023, 14 patients out of 92 (15%) underwent a hybrid revascularization strategy. Demographic and clinical characteristics of both groups are reported in detail below (Table 1). The intraoperative data are listed in Table 2. After a median follow-up of 75 months (range 3.1:149 months), cardiac mortality was 0% and overall mortality was 3%, with one case of in-hospital mortality (1%), a patient who developed a subarachnoid hemorrhage on the 5th postoperative day, and two additional late deaths. When the conversion rate to sternotomy was analyzed, two cases converted due to the intramyocardial course of the LAD were detected (2.1%). Surgical re-explorations were needed in three patients for bleeding (No bleeding was caused by having the double antiplatelets therapy (DAPT), either in patients receiving single surgical coronary bypass (isolated group) or in patients treated with a hybrid approach (hybrid group) and in two cases for revision due to graft failure (4.6%). Postoperative complications for the total MIDCAB and for the hybrid group are reported in Table 3. All patients received a LIMA to LAD grafting, and in 22 cases (24.2%), one more graft was executed. In the hybrid group, the LIMA has been used as a single graft on the LAD for every patient (Figure 2A), while in two of them, it has also been grafted to the first diagonal branch (Figure 2B). Seventy-nine patients were extubated directly in the operative room (86%) and all patients in the HCR group were extubated directly at the end of surgery (Table 3). Patients in the hybrid group underwent a two-staged PCI revascularizing a mean of 1.6 ± 0.6 vessels (Table 4). In five patients, the procedure was performed during the same hospitalization, while in another three patients, the procedure was scheduled beyond 30 days following the indexed revascularization. The follow-up was conducted until December 2023, and it was 95% complete. The mortality data rate during the follow-up was 100% complete and based on the consultation of the Swiss death bulletin (https://www.todesanzeigenportal.ch, accessed on 1 February 2024).

## 4. Discussion

Minimally invasive coronary artery bypass represents an interesting and valid option for surgical myocardial revascularization in selected patients.

This work conveys at least three relevant messages regarding minimally invasive and hybrid revascularization strategies:

a. One of the main advantages results from the avoidance of sternotomy, hence the risk for sternal wound infections (or mediastinitis) and sternal instability are completely abolished.
b. For solely surgical revascularization of the LAD by a left mini-thoracotomy, followed by staged PCI, our results demonstrate that the long-term patency rate is as good as with sternotomy.
c. Although only in a limited number of patients, the management of DAPT demonstrated a good safety profile and low bleeding risk in those patients in whom MIDCAB has been staged after PCI.

Having said that, if the association between surgery and percutaneous revascularization is adopted to achieve complete revascularization in multivessel disease, the hybrid strategy can be considered a viable one. The key question relates to the right patient selection, for whom the isolated minimally invasive or the hybrid revascularization strategy should be performed. Revising the existing literature and relative outcomes would help to answer this clinical question. One of the first large studies on MIDCAB, analyzing 300 patients, documented a short-term LIMA patency of 98% and a repeat revascularization rate of 4% [11]. In a meta-analysis by Raja et al. on about eight thousand patients, the results of LAD treatment comparing MIDCAB and PCI showed no differences in mortality, myocardial infarctions, and MACCE rate, with MIDCAB reporting a superior freedom from repeat revascularization [12]. One of the first prospective randomized trials published in 2018, the POL-MIDES Trial, compared the results of hybrid revascularization and CABG, demonstrating at 5 years follow-up in 200 patients, similar rates of myocardial infarction, repeated revascularization, and Major Adverse Cardiac and Cerebral Events (MACCE) [13]. Repossini et al., in 2019, completed a follow-up at 15 years on more than ones thousand patients who underwent MIDCAB with a survival rate of 83% for the treatment of single-vessel disease by LIMA to LAD. Looking at these data, the isolated MIDCAB can be considered a safe and effective operation [14].

There are only two reports in the literature documenting worse outcomes of hybrid revascularization. Both of them have some flaws, either the limited number of patients or the retrospective nature coupled with relatively short follow-up. Namely, a recent one showed worse outcomes in the hybrid group at two years follow-up: Hannan et al. analyzed retrospective data from New York registries. At six years follow-up, a worse survival rate (80.9% vs. 85.8%) and repeated revascularization rate (88.2% vs. 76.6%) in the hybrid arm emerged [15]. This evaluation highlighted the very low percentage of hybrid cases, 0.8% of the total CABG procedures [16,17,18,19,20]. Also, in our limited series, the patients treated with the hybrid strategy represent 1.5% of the total number of CABG patients in the same period of observation. In the STS Database, the hybrid approach only represents 0.48% of the total CABG operations in North America between 2011 and 2013 [21]. Surgical advances coupled with improvements in coronary stents may broaden the application of hybrid strategies in the future.

Other recent trials that compared the results of hybrid, standard CABG, and PCI showed an incomplete revascularization rate of 7.7%, 8.0%, and 5.7% and a restenosis rate of 8.2%, 20.4%, and 5.9%, respectively. The latter despite less residual ischemia at 12 months in the hybrid group (6.4% vs. 6.7% vs. 7.9%, respectively) [21,22,23,24].

A meta-analysis comparing a hybrid coronary revascularization and PCI found a lower rate of myocardial infarction and target vessel revascularization in the hybrid group, but no difference in mortality and stroke [25]. The same results were found in a more recent study by Patel et al., where 158 matched pairs with LAD proximal complex stenosis MIDCAB and DES-PCI have equivalent nine-years survival but PCI was associated with more frequent late reinterventions [19].

In the case of two-vessel disease or multi-vessel disease, the heart team cooperation plays a fundamental role as well as the patient preference. Indeed, when the hybrid approach is considered, key aspects to be taken into account are:

- At least a left ventricular systolic function (LVEF) > 45%.
- Good lung function because of the prolonged single lung ventilation required by this minimally invasive operation.
- The risk of an intramyocardial course of the LAD and the consequent possible implication of a sternotomy conversion.
- The management of double antiplatelet therapy (DAPT), with bleeding implications after a staged surgery.

In 2014, Gasior et al. conducted a feasibility study in a cohort of two hundred patients who had undergone hybrid revascularization in a two-stage fashion; the PCI was performed after 24 h from the MIDCAB operation. They observed a sternotomy conversion of 6.1%, but no major bleeding complications (2.0%) [20]. More recently, the HYBRID-COR feasibility study based on a concomitant procedure by means of Endoscopic Coronary Artery Bypass (ECAB) and PCI on DAPT in a hybrid operating room on 30 patients showed only one chest revision for bleeding and one death at more than 4 years follow-up [22]. The management of DAPT represents a key point in the hybrid strategy for myocardial revascularization. In our hybrid series, 5 out of 14 patients underwent PCI during the same hospitalization but in a two-stage fashion, not concomitantly, without facing bleeding complications that required surgical revision. Indeed, the optimal treatment protocols are still debated: two-stage coronary revascularization ensures optimal results and does not require a hybrid operating room. However, they could face a risk of ischemia originating from non-LAD territories during the surgical grafting and a cumulative risk (negligible) of repeated reintervention if the percutaneous approach fails. On the other hand, patients undergoing PCI before surgery could have a risk of stent thrombosis, increased peri- and post-operative bleeding due to dual antiplatelet therapy (although this issue was satisfactory in our series, thanks to careful Multiplate analysis and Cangrelor Embrication), along with complications in the LAD territory during the separating time interval [23]. Last, the anastomosis is not evaluated in angiography if the PCI is performed as the first step. Although with limited evidence [24], all these issues could be overcome with a one-stage hybrid procedure protocol. The procedure requires that the hybrid room and the entire operating team must be experienced, ensuring that the PCI to high-risk non-LAD lesions can be safely performed with a protected anterior territory, and conventional CABG remains an option in cases of unsuccessful stent implantation. Moreover, surgical anastomosis can be studied before performing PCI. 

The results of the STS database reported a reoperation for bleeding in 3.6% and 2.4% for a concomitant and two-stage procedure, respectively, with a mortality rate of 3.6% vs. 1.4% [21]. Repossini et al. compared the rate of reoperation for bleeding in the single MIDCAB group and a two-stage hybrid group and it resulted in 1.5% and 2.5%, respectively; a bleeding amount of more than 1000 mL that did not require reoperation was remarkable too (1.2% vs. 4.5%) [18]. The MERGING trial that compared 40 hybrids with a PCI performed 48–72 h after operation, and 20 CABG patients showed bleeding rates of 7.5% and 5.0%, respectively, although this was not statistically significant [19].

Actually, there is no evidence in those limited reports in the literature that HCR provides better results than traditional CABG or PCI in multivessel disease, and for this reason, there are nowadays no guidelines. We will be able to improve the results with the use of multiple arterial grafts and next-generation stents. A hybrid approach can be considered a viable option in myocardial revascularization when solid cooperation between cardiologists and cardiac surgeons is established, with careful attention to indications, limitations, and management, because not every patient is suitable for HCR. An ongoing multicenter randomized trial will confirm the quality of life and recovery between MIDCAB and standard sternotomy CABG the MIST Trial [25]. This study will eventually corroborate the very good outcomes of Mid-CAB in the long term (more than 20 years) when performed in a carefully selected patient population [26,27,28]. Robotic iterations of this operation are already performed in leading centers worldwide, aiming to establish the maximal level of a true minimally invasive approach and providing good long-term outcomes in patients who are not candidates for conventional CABG [29].

## 5. Study Limitations

The study has some drawbacks to disclose. First, it is a single-center feasibility report, without a control group (the HCR group separately reports its outcomes in a longitudinal way, within the isolated overall MIDCAB, not as a comparative analysis) and the analyzed sample is relatively small. Although all the HCR patients underwent a control coronary angiography, no routine coronary angiography was performed in asymptomatic patients treated with isolated MIDCAB during follow-up.

## 6. Conclusions

Isolated MIDCAB and hybrid revascularization are associated with encouraging short- and long-term results in appropriately selected patients. All these evaluations should be made within the heart team based on clinical presentation and on individual coronary anatomy. The staging of the indexed procedure can differ and a certain flexibility concerning the technical variables is advisable given the good surgical outcomes when a double antiplatelet therapy is adopted. Complete revascularization should be the cornerstone independent of the strategy adopted.

## Figures and Tables

**Table 1:** Demographic and clinical characteristics.

	N (Percentage %)	
	Tot. MIDCAB	Hybrid
Patients	92 (100%)	14/92 (1.5%)
Age (years)	67.9 ± 10.4	72 ± 10.5
Male	81 (88%)	12 (86%)
Smoking	23 (25%)	4 (29%)
Hypertension	64 (70%)	13 (93%)
Dyslipidemia	61 (66%)	9 (64%)
Chronic coronary syndrome	25 (27%)	2 (14%)
Unstable angina	67 (73%)	12 (86%)
History of myocardial infarction	26 (28%)	2 (14%)
Diabetes type I under insulin therapy	13 (14%)	1 (7%)
Diabetes type II	28 (30%)	4 (29%)
PAD	7 (8%)	0 (0%)
Number of diseased vessels (N, %)	1 vessel: 33 (36%) 2 vessels: 47 (51%) 3 vessels: 12 (13%)	1 vessel: 0 (0%) 2 vessels: 9 (64%) 3 vessels: 5 (36%)
Euroscore II	1.0 ± 0.7	0.95 ± 0.5
Ejection Fraction (%)	56 ± 7.5	53 ± 6.2

Data are expressed as mean value ± standard deviation (SD). PAD: Peripheral Artery Disease.

**Table 2:** Intraoperative data.

	N (Percentage %)	
	Tot. MIDCAB	Hybrid
N° of bypass		
1	58 (63%)	12 (86%)
2	30 (33%)	2 (14%)
3	4 (4%)	0 (0%)
Lima	92 (100%)	14 (100%)
+ Rima	4 (4%)	0 (0%)
+ Radial	1 (1%)	0 (0%)
+ SVG	17 (18%)	0 (0%)
Op. time (min)	210 ± 85	178 ± 65
CPB time in 7 cases (min)	117 ± 41	0 (0%)
X-Clamp time in 2 cases (min)	62	0 (0%)

Data are expressed as mean value ± standard deviation (SD). Lima: Left Internal Mammary Artery; Rima: Right Internal Mammary Artery; SVG: Saphenous Vein Graft; CPB: Cardiopulmonary Bypass.

**Table 3:** Postoperative Complications.

	N (Percentage %)	
	Tot. MIDCAB	Hybrid
30-day Mortality	1 (1%)	0
Extubation		
POD 0	79 (86%)	14 (100%)
POD 1	11 (12%)	0 (0%)
POD > 1	2 (2%)	0 (0%)
Graft failure	2 (2.1)	0 (0%)
Sternotomy conversion	2 (2.1)	0 (0%)
Surgical revision	3 (3.2)	0 (0%)
ICU stay (days)	1.3 ± 0.7	1
Post-operative Hospital stay (days)	6.4 ± 3.9	6.3 ± 1.3
Long-term Mortality	2 (2.1%)	0 (0%)

Data are expressed as mean value ± standard deviation (SD). POD: Post-Operative Day; ICU: Intensive Care Unit.

**Table 4:** Hybrid coronary revascularization strategy.

	Hybrid Coronary Revascularization (14 Patients)
N° of treated vessels (out of LAD)	1.6 ± 0.6
N° of treated vessels (out of LAD) per patient (N, %)	
- 1 vessel	6/14 (43%)
- 2 vessels	7/14 (50%)
- 3 vessels	1/14 (7%)
N° of Drug Eluting Stent (mean ± SD)	2.1 ± 0.9
Type of Drug Eluting Stent (target vessels)	ORSIRO (RCA) BIOFREEDOM (RCA) XIENCE SIERRA (RCA and CX) ULTIMASTER TANSEI (RCA and CX) ORSIRO MISSION (RCA and RCX) XIENCE SKYPOINT (RCA) RESOLUTE ONYX (PDA) ONYX FRONTIER (RCA and PLA) ULTIMASTER NAGOMI (RI)
PCI before Surgery (n° of pts, %)	4 (28%)
Days before Surgery (mean ± SD)	28 ± 21
PCI after Surgery (n° of pts, %)	10 (72%)
Days after Surgery (mean ± SD)	31 ± 24
LIMA angiographic patency at staged PCI (n° of pts, %)	10 (100%)
PCI during the same hospitalization (n° of pts, %)	5 (36%)
Re-exploration for bleeding due to DAPT	0 (0%)

LAD: left anterior descending; PCI: percutaneous coronary revascularization; DAPT: dual antiplatelet therapy; SD: standard deviation; RCA: right coronary artery; PDA: posterior descending artery; CX: circumflex artery; PLA: posterolateral artery; RI: ramus intermedius.

## Data Availability

Available by request to the Authors.
